# Lipid Composition of Latex and Rubber Particles in *Hevea brasiliensis* and *Taraxacum kok-saghyz*

**DOI:** 10.3390/molecules25215110

**Published:** 2020-11-03

**Authors:** Sung Woo Bae, Sunghee Jung, Sang Chul Choi, Mi Young Kim, Stephen Beungtae Ryu

**Affiliations:** 1 Plant Systems Engineering Research Center, Korea Research Institute of Bioscience & Biotechnology (KRIBB), Daejeon 34141, Korea; cantabile531@gmail.com (S.W.B.); shjung89@kribb.re.kr (S.J.); choisc.wiseman@gmail.com (S.C.C.); mykim3890@kribb.re.kr (M.Y.K.); 2 Department of Biosystems and Bioengineering, KRIBB School of Biotechnology, University of Science and Technology (UST), Daejeon 34141, Korea; 3 Research & Development Center, JICO Ltd., Daejeon 341486, Korea

**Keywords:** natural rubber, Pará rubber tree, rubber dandelion, phospholipid, galactolipid

## Abstract

Natural rubber is usually synthesized in the rubber particles present in the latex of rubber-producing plants such as the Pará rubber tree (*Hevea brasiliensis*) and rubber dandelion (*Taraxacum kok-saghyz*). Since the detailed lipid compositions of fresh latex and rubber particles of the plants are poorly known, the present study reports detailed compound lipid composition, focusing on phospholipids and galactolipids in the latex and rubber particles of the plants. In the fresh latex and rubber particles of both plants, phospholipids were much more dominant (85–99%) compared to galactolipids. Among the nine classes of phospholipids, phosphatidylcholines (PCs) were most abundant, at **~**80%, in both plants. Among PCs, PC (36:4) and PC (34:2) were most abundant in the rubber tree and rubber dandelion, respectively. Two classes of galactolipids, monogalactosyl diacylglycerol and digalactosyl diacylglycerol, were detected as 12% and 1%, respectively, of total compound lipids in rubber tree, whereas their percentages in the rubber dandelion were negligible (< 1%). Overall, the compound lipid composition differed only slightly between the fresh latex and the rubber particles of both rubber plants. These results provide fundamental data on the lipid composition of rubber particles in two rubber-producing plants, which can serve as a basis for artificial rubber particle production in the future.

## 1. Introduction

The Pará rubber tree (*Hevea brasiliensis*, Hevea) has been the sole source of natural rubber for a long time. These trees are mostly cultivated in countries of the Asia-Pacific region; therefore, the natural rubber used worldwide is primarily produced here [1]. However, this restricted production gives rise to risk factors that unbalance the supply of natural rubber. Plant diseases such as South American leaf blight can exterminate rubber trees [2]. Moreover, climate change has threatened the survival of endemic rubber trees, such as those growing in the tropics [3,4]. In addition, the increasing demand for natural rubber worldwide has rendered adequate supply even more difficult [5,6].

The Russian dandelion (*Taraxacum kok-saghyz*, TKS), which has lately been called the rubber dandelion, has been studied as a major alternative crop since World War II, because it can produce natural rubber of good quality [1,4]. Although the rubber polymer of TKS is longer than that of Hevea, its rubber productivity per acre is lower [1,7,8]. With the improvement of its rubber productivity, TKS may be utilized as an alternative rubber crop to stabilize the global supply of natural rubber [1,9].

Both Hevea and TKS have specialized natural rubber-producing tissues called laticifers [4]. Laticifers store latex [10] with numerous particles with a lipid monolayer structure called rubber particles, which store natural rubber [11]. The rubber particles isolated from latex and washed with buffer are called washed rubber particles [12]. Many proteins are attached to the lipid membrane of rubber particles, and some of these proteins are involved in natural rubber synthesis.

The biosynthesis of natural rubber has been suggested to be catalyzed by rubber transferase complex bound to rubber particles [1]. The identification of a rubber transferase complex including *cis*-prenyltransferase would help to elucidate the mechanism of natural rubber synthesis, and extensive research on this topic is underway worldwide [13,14,15]. In the studies, to confirm their activity, proteins attached to the rubber particles were washed; candidate proteins were recombined with washed rubber particles; and the rubber synthesis capacity of the recombined rubber particles was measured. However, the major shortcoming of functional studies is the low reliability of experimental results regarding a specific candidate rubber transferase complex, as all proteins cannot completely be washed from the rubber particles [13,15]. Therefore, clean rubber particles without any proteins or with only structural proteins are required to obtain accurate results. In this context, the knowledge of lipid composition of rubber particles in natural rubber-producing plants would aid the synthesis of artificial rubber particles, which would further help identify rubber transferase complex and rubber biosynthesis-related proteins. Moreover, artificial rubber particles may be used for in vitro rubber polymer biosynthesis in the future.

There have been several studies on the lipid composition of latex and rubber particles in Hevea; however, most studies focused on processed latex or did not take the problem of rapid degradation of compound lipids into account [16,17,18,19,20,21,22]. Moreover, little is known about the lipid composition of fresh latex and rubber particles in TKS [23]. Therefore, in this study, the compound lipid compositions of fresh latex and rubber particles in Hevea, as well as TKS, were analyzed together after addressing the problem of rapid lipid degradation and compared with previous reports obtained from mostly Hevea. In addition, compound lipid compositions of fresh latex and rubber particles were compared between Hevea and TKS. The detected compound lipids included phospholipids and galactolipids, which are the major lipid components of intracellular membranes.

## 2. Results

### 2.1. Extraction of Lipids from the Latex and Rubber Particles of Hevea and TKS

**Figure 1:**
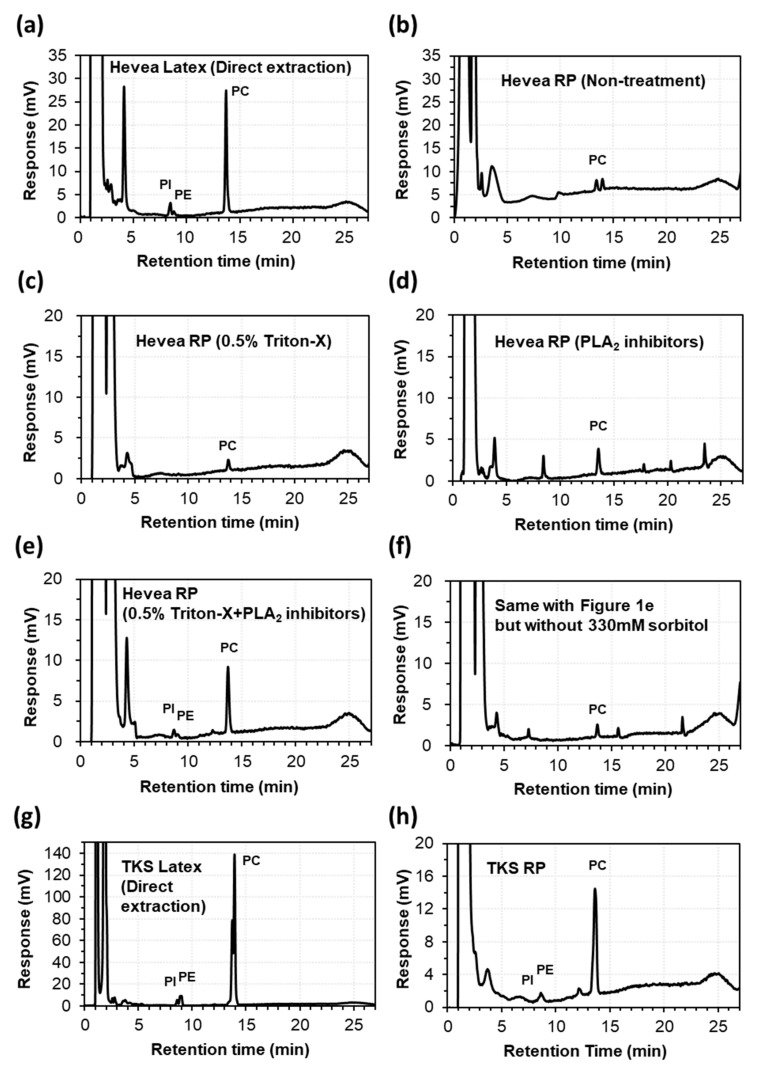
HPLC-ELSD chromatogram of phospholipids in the latex and rubber particles (RP) of *Hevea brasiliensis* (Hevea) and *Taraxacum kok-saghyz* (TKS). (**a**) Hevea latex (direct extraction); (**b**) Hevea RP (non-treatment); (**c**) Hevea RP (0.5% Triton-X); (**d**) Hevea RP (phospholipase A (PLA) inhibitors); (**e**) Hevea RP (0.5% Triton X + PLA inhibitors); (**f**) same with Figure 1e but without 330 mM sorbitol; (**g**) TKS latex (Direct extraction); (**h**) TKS RP (0.5% Triton X + PLA inhibitors).

In HPLC-ELSD analysis, the lipid extract of fresh Hevea latex showed a clear major peak corresponding to phosphatidylcholines (PCs), as well as minor peaks corresponding to phosphatidylinositols (PIs) and phosphatidylethanolamines (PEs) (Figure 1a), as compared to the standard lipids (Supplementary Materials Figure S1). However, the lipid extracts of washed rubber particles showed a dramatic suppression or disappearance of the PC, PI, and PE peaks when the latex collection solution was supplemented without (Figure 1b) or with 0.5% Triton-X (Figure 1c). In a previous study [21], high lysophosphatidylcholine (LPC) content was detected in processed latex of Hevea following harvest, indicating strong activity of phospholipase A (PLA), which hydrolyzes PC to generate LPC. Therefore, a mixture of PLA inhibitors was added to the latex collection solution and washed rubber particles were prepared, followed by immediate lipid extraction. PC hydrolysis was reduced when the latex collection solution was supplemented with PLA inhibitors (Figure 1d) and even more clearly when supplemented with PLA inhibitors plus 0.5% Triton-X (Figure 1e).

When 330 mM sorbitol was removed from the latex collection solution of Figure 1e, PC was hydrolyzed, as evidenced by increased LPC content, indicating that 330 mM sorbitol is also important to protect lipids from degradation during the preparation of washed rubber particles (Figure 1f). In test experiments of sample preparation, lipid extraction was performed according to the protocol described in a previous study [24]. According to this protocol, the chloroform and aqueous phases are not separated during incubation for lipid extraction, which might lead to greater lipid degradation by PLA.

To reduce the contact between lipids and endogenous PLA, lipids were extracted using modified Folch’s protocol, in which the two phases are separated and mixed only during vortexing. HPLC-ELSD analytical results for TKS latex and rubber particles are presented in Figure 1g,h, respectively. The samples for further lipid analysis using electrospray ionization and triple quadrupole tandem mass spectrometry (ESI-MS/MS) was prepared using the modified Folch’s protocol with a shortened incubation time and small amounts of samples, which was described in Materials and Methods.

### 2.2. Lipid Composition

**Figure 2:**
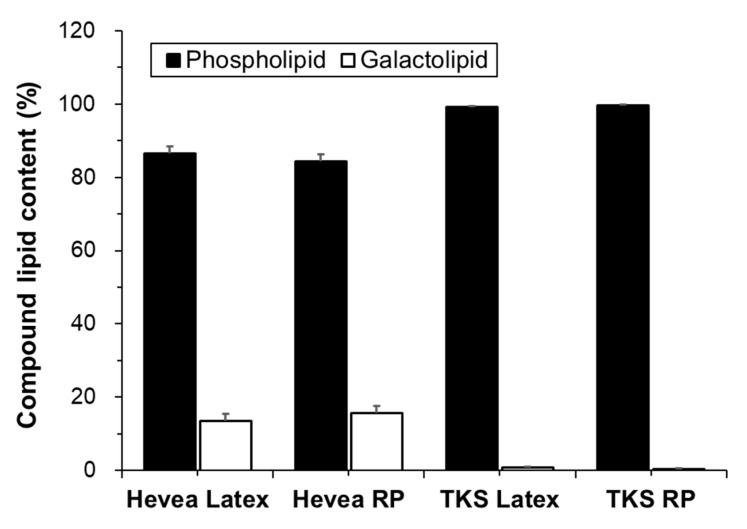
Contents of phospholipids and galactolipids in the latex and rubber particles (RP) of Hevea and TKS (mol% of total compound lipid).

Variations in the proportions of phospholipids and galactolipids in the fresh latex and rubber particles of Hevea and TKS are shown in Figure 2. Phospholipids were greatly more abundant (> 85% of total compound lipids) than galactolipids in the fresh latex and rubber particles of both plants. Galactolipids were detected at trace amounts (<1% of total compound lipids) in TKS compared to the amounts detected in Hevea (15% of total compound lipids). On the contrary to our study, however, most previous studies have reported glycolipid (galactolipid) contents similar to or higher than phospholipid contents in Hevea latex or rubber particles [17,18,21,24,25]. In our study, the lipid compositions of latex and rubber particles differed only slightly in both plants tested, indicating that most lipids in the latex are produced from rubber particles.

#### 2.2.1. Phospholipids

**Figure 3:**
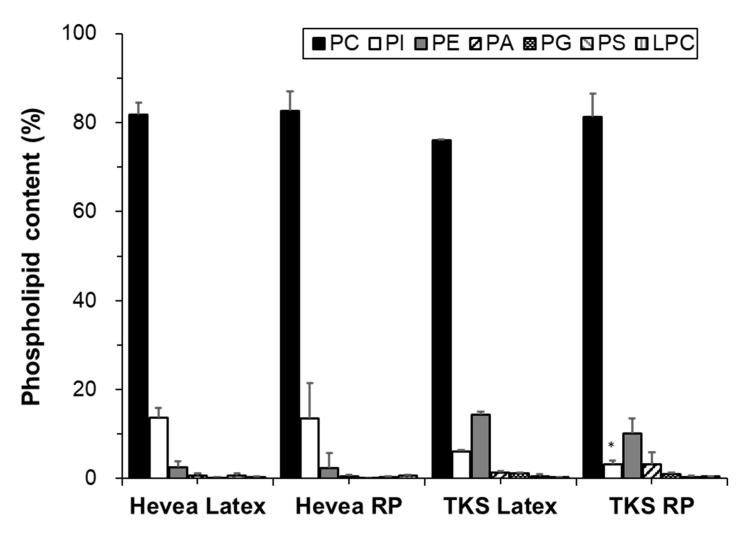
Contents of phospholipids in the latex and rubber particles (RP) of Hevea and TKS (mol% of total phospholipid). The asterisks indicate statistically significant differences (latex versus RP) as determined by *t*-tests: * *p* < 0.01.

Phospholipids contain two fatty acids and one phosphate group bound to glycerol [26]. In our analyses, the following phospholipids were identified in the latex and rubber particles of both Hevea and TKS: PC, PI, PE, phosphatidic acid (PA), phosphatidylglycerol (PG), phosphatidylserine (PS), lysophosphatidylcholine (LPC). The phospholipid classes and their composition in each sample are shown in Figure 3. The identified phospholipids were divided into three groups according to their proportions. In group I, PCs were particularly abundant in Hevea and TKS, at ~80% of total phospholipids. In group II, PI and PE accounted for ~2–14% of total phospholipids in both plants. In Hevea, PI was more abundant than PE (13% and 6% of total phospholipids, respectively), whereas in TKS, PE was more abundant than PI (11–14% and 2% of total phospholipids, respectively). In group III, other phospholipids, including PA, PS, PG, and LPC, were detected at trace levels (≤1%).

Our results are consistent with those of previous studies in which PC was the most abundant phospholipid in Hevea latex [16,17,18,19,21]. However, significantly higher amounts of LPC and PA have been reported in the previous studies with Hevea latex, indicating the degradation of PC and other phospholipids [21,27].

##### Group I: Phosphatidylcholines

The major PCs detected in each sample are presented in Table 1. In Hevea, PC (36:4) showed the highest content (~30% of total PCs), followed by PC (36:2), PC (36:3), and PC (34:2) (10–20% of total PCs), in both latex and rubber particles. These results are similar to the PC composition of Hevea clone RRIM600, in which PC (36:4) is the major component, followed by PC (36:2). The trend of that PC (36:4), PC (36:3), and PC (36:2) were the major PCs in Hevea in this study is consistent with the PC composition of Hevea clone BPM24, although the actual percentages are different [21].

In contrast, in TKS, PC (34:2) showed the highest content (~50% of total PCs), followed by PC (36:4) (~20% total PCs). Our results are consistent with those of previous report in *T. brevicorniculatum* [23]. PC (34:3) accounted for < 10% of total PCs.The lipid composition of latex and rubber particles slightly differed in both the two plants studied, because latex contains rubber particles as well as other intracellular membranes. Of note, different classes of PCs were somewhat evenly distributed in Hevea, while a few specific classes of PCs were abundant in TKS.

##### Group II: Phosphatidylinositols and Phosphatidylethanolamines

Table 2 and Table 3 summarize the distributions of PI and PE classes, respectively. In Hevea, PI was the second most abundant phospholipid following PC. The most abundant PIs were PI (34:2) and PI (36:2) (~30% and ~26% of total PIs, respectively). PI (34:3), PI (36:4), and PI (36:3) accounted for ~10% of all PIs. This distribution of PIs is similar to previous reports [21]. In TKS, however, PI (34:2) alone accounted for ~80% of total PIs (Table 2). Therefore, PI composition was markedly differed between the two plants studied.

Among PEs, PE (36:2), PE (36:4), and PE (36:3) were abundant in the latex and rubber particles of Hevea. PE (34:2) was more abundant in latex, while PE (32:1) and PE (32:0) were more abundant in rubber particles (Table 3). This distribution of PEs in Hevea latex is similar to previous reports [21], although PE (36:4) was detected only in this study. Unlike that in Hevea, PE was the second most abundant phospholipid following PC in TKS. PE (34:2) and PE (36:4) accounted for respectively 40% and 30% of total PEs in both latex and rubber particles of TKS. Together, these two PEs were particularly abundant, accounting for over 70% of total PEs.

In both plants, the three classes of lipids, PC, PI, and PE, accounted for a major proportion (94–98%) of total phospholipids in the latex and rubber particles. While the lipid distribution according to the number of carbons and double bonds was relatively even in Hevea, one or two specific lipids occupied the majority in TKS.

##### Group III: Minor Phospholipids

Tables S1 to S4 (in the Supplementary Materials) present distributions of minor phospholipids, including PA, PG, PS, and LPC, respectively, which represented only 2–6% of total phospholipids.

Four classes of PA, including PA (34:2), PA (36:2), PA (36:3), and PA (36:4), were detected in Hevea. The contents of each PA were somewhat different between the latex and rubber particles (Table S1). PA (36:3) and PA (36:2) contents were similar to previous reports [21]; however, PAs with 38 carbons were not identified but PAs with 34 or fewer carbons were detected for the first time in the present study. In TKS, PA (34:2) accounted for respectively 63% and 52% of all PAs in latex and rubber particles, indicating that only one class was highly abundant.

Among PGs, PG (34:2) was the most abundant class in the latex and rubber particles of both plants, although its contents differed between them (Table S2). PG (34:2) accounted for respectively 55% and 90% of total PGs in Hevea and TKS. Of note, only one class of PG was dominant in the latex and rubber particles of TKS.

Among PSs, PS (38:2) was the most abundant, followed by PS (36:2) and PS (40:2), in Hevea (Table S3). In contrast, PS (34:2) was the most abundant, followed by PS (36:4), PS (38:2), and PS (40:2), in TKS.

LPC (18:2) accounted for ~50% of total LPCs in the latex and rubber particles of both plants (Table S4). LPC (18:1) and LPC (16:0) were detected in Hevea and TKS, respectively. The proportions of LPC (18:2), LPC (18:1), and LPC (18:0) in Hevea latex were similar to previous reports [21].

#### 2.2.2. Galactolipids

**Figure 4:**
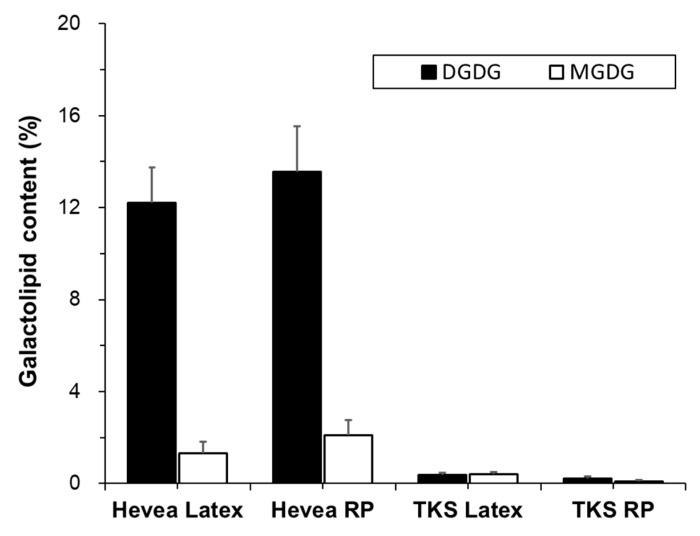
Contents of digalactosyl diacylglycerols (DGDG) and monogalactosyl diacylglycerols (MGDG) in the latex and rubber particles (RP) of Hevea and TKS (mol% of total compound lipid).

Monogalactosyl diacylglycerols (MGDGs) and digalactosyl diacylglycerols (DGDGs) are galactolipids, a type of glycolipid, in which lipids are attached to galactose. These lipids are mainly found in the chloroplast membrane of plants [20,28]. The proportions of galactolipids in Hevea and TKS are presented in Figure 4.

DGDG was the major galactolipid in Hevea (12% and 13% of total compound lipids in latex and rubber particles, respectively), while MGDG was detected at trace amounts (1% and 2% of total compound lipids in latex and rubber particles, respectively). This distribution is similar to previous reports [17,20]. Both galactolipids were detected at trace amounts (<1%) in the latex and rubber particles of TKS.

##### Digalactosyl Diacylglycerols

Major DGDGs in each sample are listed in Table 4. DGDG (36:4), DGDG (36:3), and DGDG (36:2) individually accounted for 20% of all DGDGs in Hevea latex and rubber particles. DGDG (36:1) accounted for ~10% of all DGDGs. These results are similar to previous reports in Hevea latex [20]. Five DGDGs, namely DGDG (36:5), DGDG (36:4), DGDG (36:6), DGDG (34:3), and DGDG (34:2), were the major components of TKS latex and rubber particles.

##### Monogalactosyl Diacylglycerols

MGDG classes in each sample are listed in Table 5. MGDG (36:4), MGDG (36:3), and MGDG (36:2) were the major classes in the latex and rubber particles of Hevea. This distribution is comparable to previous reports [20]; however, the most abundant MGDG was MGDG (36:4) in our study and MGDG (36:2) in a previous study [20].

MGDG (36:5), MGDG (36:4), and MGDG (36:6) were the major classes in the latex and rubber particles of TKS. Since TKS galactolipids were detected at trace amounts, actual contents of individual classes in the latex and rubber particles of TKS were negligible.

## 3. Discussion

Latex contains numerous rubber particles, whose membranes comprise a lipid monolayer. Phospholipids constitute the skeleton of the cellular lipid membrane [29]. Accordingly, all samples in the present study showed a greatly higher percentage of phospholipids than that of galactolipids. Typically, PCs and PEs are the most abundant phospholipids in the cellular lipid membranes of plants [30]. Of note, however, PCs accounted for a markedly high percentage of total phospholipids in rubber particles in the latex of rubber tree and rubber dandelion in the present study (Table 6).

In previous studies, PCs and LPCs were reported to be the major phospholipid classes detected in Hevea latex samples [21,27] and PCs in *T. brevicorniculatum* rubber particles [23]. LPCs may be generated as byproducts due to the activities of PLA, possibly during tapping and extraction processes [21]. During the processing of Hevea latex, phospholipids are degraded more rapidly than glycolipids, mainly galactolipids [21]. Thus, during the storage of Hevea latex or rubber particles, the percentage of phospholipids is initially higher but subsequently lower than that of glycolipids [21,25]. Higher amounts of glycolipids than those of phospholipids have also been reported in other studies [17,18,24]. To the best of our knowledge, however, no previous study has addressed the prevention of lipid degradation during rubber particle isolation.

Contrary to the previous reports that LPCs and PAs showed somewhat higher amounts [21], these two lipids were detected at trace amounts in the present study. This result may be attributed to the suppression of PLA-mediated phospholipid degradation during the extraction of latex and rubber particles; this was achieved by adding 330 mM sorbitol and PLA inhibitors plus 0.5% Triton-X to the latex collecting buffer and applying the modified Folch’s protocol using shortened extraction time and small amounts of samples.

Both Hevea and TKS are natural rubber-producing plants; however, climatic conditions in regions where they grow vary widely [4]. Unlike rubber dandelions that grow in a continental climate [31,32], Hevea mainly grows in tropical regions including Southeast Asia, where both temperature and rain are high [33]. Therefore, cells and tissues of Hevea that are exposed to a hot environment may be differently adapted to tolerate stress, and natural rubber production is presumed to be one of such adaptations. In fact, there is evidence that Hevea uses natural rubber to protect itself from hot and wet environments [34]. Rubber particles have been speculated to be synthesized by intracellular endoplasmic reticulum [35]. Rubber particles are the sites where natural rubber is synthesized and stored in latex. Lipid composition of rubber particles may be affected by the intracellular environment that is optimized for stress resistance. The higher proportions of galactolipids, particularly DGDGs, in Hevea than in TKS indicate that galactolipids, such as DGDGs, likely play important roles in thermo-tolerance, as suggested in a previous study [36].

The number of double bonds in lipids affects the fluidity of the lipid membrane. Changes in the number of double bonds alter the membrane structure. Unsaturated lipids having one or multiple double bonds result in increased membrane fluidity [37]. Plants need to adapt to the surrounding environment for survival, and appropriate changes in membrane fluidity are essential for adaptation to hot or cold environments [38]. According to previous studies, exposure to extreme temperatures increased the degree of unsaturation of lipids constituting the membrane, making it more fluid [39,40]. Therefore, the difference in lipid composition in terms of the number of carbons and double bonds between Hevea and TKS plants is considered to reflect the adaptive strategies of plants to the environments they inhabit.

## 4. Materials and Methods

General chemicals and compound lipids were purchased from Sigma-Aldrich (St. Louis, MO, USA), unless otherwise stated.

### 4.1. Collection of Latex and Preparation of Washed Rubber Particles

Latex was collected from Pará rubber tree and rubber dandelion (USDA accession no. W6 35166), which were grown in a greenhouse located at KRIBB, Daejeon, South Korea. Pará rubber tree was scratched with a knife [12] and ~100 μL of latex was collected with a pipette. The thick part between the leaf and roots of rubber dandelion was cut, and ~100 μL of flowing latex was collected from three different plants. The collected latex samples were immediately added to 10× volume of chloroform/MeOH (2:1) containing 0.002% BTH and subjected to lipid extraction, as described below.

To prepare washed rubber particles, 100 μL of latex was collected in 1.5 mL of ice-chilled buffer solution [10 mM sodium phosphate (pH 7.5), 1 mM EGTA, 2 mM DTT, and 330 mM sorbitol] in a 2 mL Eppendorf tube. After centrifugation of the latex-suspended buffer at 27,000× *g* for 15 min at 4 °C, the rubber particles floating in the supernatant were transferred to a new Eppendorf tube and suspended in 1 mL of ice-chilled fresh buffer. The process was repeated twice, resulting in the preparation of washed rubber particles. If necessary, to suppress PLA activity, 0.5% Triton-X and a mixture of PLA inhibitors (3 µM manoalide, ONO, BEL, and AACOCF3) [41] were added to the latex collection solution.

### 4.2. Lipid Extraction from Latex and Rubber Particles

Unless otherwise stated, the lipids were extracted according to Folch’s protocol [42] with some modifications. Briefly, the latex and rubber particle suspensions were added to 10× volume of chloroform/MeOH (2:1) containing 0.002% BTH. The mixtures were vortexed vigorously for 5 min, and 1/10th volume of 2M KCl was added. The mixture was vortexed again and then centrifuged at 6,500× *g* for 5 min at 4 °C. The bottom phase (chloroform and MeOH) was transferred into a new tube and dried with N_2_ gas.

### 4.3. HPLC-ELSD Analysis

The extracted lipids were first analyzed using HPLC-ELSD to monitor phospholipid degradation. Dried lipid extracts were dissolved in chloroform/methanol (95:5) and injected into a UHPLC system (Shimadzu, Kyoto, Japan) equipped with a Rheodyne manual injector with a 20 μL sample loop and a silica column [Shim-pack CLC-SIL (M); 4.6 mm i.d. × 15 cm, Shimadzu]. Chromatographic separation was performed using a linear binary gradient according to the following scheme: t_0_: 100%A, 0%B; t_7_: 80%A, 20%B; t_12_: 75%A, 25%B; t_17_: 75%A, 25%B; t_22_: 100%A, 0%B, and t_27_: stop. The total chromatographic run time was 32 min per sample, including a 27 min analysis and 5 min re-equilibration. Eluent A comprised chloroform/methanol/ammonium hydroxide (800:225:5), and eluent B comprised methanol/water/ammonium hydroxide (800:190:5). The flow rate of the eluent was 1.0 mL·min^−1^. An evaporative light scattering detector (SEDEX Model 80 LT-ELSD) was used; the pressure of nebulizer nitrogen gas was maintained at 350 kPa, and the drift tube temperature was set at 50 °C.

### 4.4. Lipid Analysis Using ESI-MS/MS

After quality check, the lipid samples that did not show evident phospholipid degradation were quantified using ESI-MS/MS at the Kansas Lipidomics Research Center, Kansas State University [43], which is supported by NSF, Kansas Technology Enterprise Corporation, K-IDeA Networks of Biomedical Research Excellence of NIH, and Kansas State University. Unfractionated extracts were directly injected into a mass spectrometer and subjected to ESI-MS/MS in the precursor and neutral loss scanning modes to identify polar lipids. The identified lipids were quantified with reference to two internal standards for the different lipid classes using a correction curve and expressed in mol% as previously described [43].

### 4.5. Statistical Analysis

All data are expressed as the mean value of at least three biological replicates. The data were analyzed using *t*-tests, and differences were considered statistically significant for a *p*-value of < 0.05.

## 5. Conclusions

In this study, compound lipid compositions of the latex and rubber particles in Hevea and TKS were examined, compared and summarized in Table 6. This study provides fundamental data for the production of artificial rubber particles, which can be further used to elucidate the functions of rubber particle proteins, such as rubber transferase complex, involved in natural rubber synthesis. However, since additional substances, such as other lipids and structural proteins, constitute rubber particles, additional research is warranted to elucidate these components. By identifying rubber transferase candidates through further research, artificial rubber particles can be synthesized and utilized for the functional assays of rubber biosynthesis. This system may also be further developed for in vitro bio-rubber production.

## Figures and Tables

**Table 1:** Classes and proportions of phosphatidylcholines in each sample (mol%).

		Hevea			TKS	
Mass	PC	Latex	WRP		Latex	WRP
756.5	PC (34:3)	2.69 ± 0.25	3.53 ± 0.70		12.63 ± 1.77	11.30 ± 0.89
758.6	PC (34:2)	9.11 ± 0.96	10.94 ± 1.66		53.43 ± 3.02	50.08 ± 1.30
760.6	PC (34:1)	2.80 ± 0.37	3.35 ± 0.57		2.59 ± 1.22	3.24 ± 2.22
780.5	PC (36:5)	4.73 ± 0.80	5.09 ± 1.84		6.95 ± 1.35	7.46 ± 1.24
782.6	PC (36:4)	30.89 ± 7.58	27.01 ± 10.12		18.51 ± 3.46	21.23 ± 3.07
784.6	PC (36:3)	17.70 ± 2.25	17.20 ± 3.48		2.13 ± 0.49	3.05 ± 1.46
786.6	PC (36:2)	19.75 ± 1.57	18.62 ± 3.07		0.69 ± 0.05	0.87 ± 0.14
788.6	PC (36:1)	61.7 ± 1.22	5.43 ± 2.68		0.02 ± 0.04	0.05 ± 0.03
810.6	PC (38:4)	1.80 ± 2.26	3.83 ± 2.70		0.10 ± 0.02	0.07 ± 0.02
812.6	PC (38:3)	1.35 ± 1.14	1.67 ± 0.88		0.12 ± 0.03	0.11 ± 0.01

The PC species of which the proportions were less than 1% were removed (PC 32:0, PC 34:4, PC 36:6, PC 38:2, PC 38:5, PC 38:6, PC 40:2, PC 40:3, PC 40:4, PC 40:5).

**Table 2:** Classes and proportions of phosphatidylinositols in each sample (mol%).

		Hevea			TKS	
Mass	PI	Latex	WRP		Latex	WRP
824.50	PI (32:2)	0.33 ± 0.00	0.31 ± 0.06		1.20 ± 0.32	0.98 ± 0.65
850.50	PI (34:3)	8.62 ± 0.52	9.35 ± 2.62		8.21 ± 0.55	9.12 ± 1.89
852.50	PI (34:2)	33.69 ± 6.70	29.88 ± 2.93		82.27 ± 0.70	77.41 ± 1.85
854.50	PI (34:1)	5.45 ± 0.75	5.10 ± 0.34		1.06 ±0.42	2.17 ± 1.74
874.50	PI (36:5)	2.30 ± 0.31	2.29 ± 0.69		1.07 ± 0.44	1.46 ± 0.62
876.50	PI (36:4)	9.84 ± 1.28	9.33 ± 2.54		3.42 ± 1.04	4.36 ± 0.97
878.50	PI (36:3)	10.84 ± 4.89	12.80 ± 3.22		0.70 ± 0.03	1.56 ± 0.59
880.60	PI (36:2)	24.67 ± 2.55	26.21 ± 1.52		1.65 ± 0.17	2.52 ± 0.85
882.60	PI (36:1)	3.37 ± 0.81	3.87 ± 1.63		0.00 ± 0.00	0.14 ± 0.15

The PI species of which the proportions were less than 1% were removed (PI 32:0, PI 32:1, PI 32:3, PI 34:4, PI 36:6).

**Table 3:** Classes and proportions of phosphatidylethanolamines in each sample (mol%).

		Hevea			TKS	
Mass	PE	Latex	WRP		Latex	WRP
688.5	PE (32:2)	1.20 ± 0.45	0.74 ± 0.76		3.97 ± 1.21	3.01 ± 1.83
690.5	PE (32:1)	5.84 ± 4.88	12.42 ± 10.02		0.09 ± 0.08	0.09 ± 0.09
692.5	PE (32:0)	1.66 ± 0.95	10.62 ± 9.92		0.03 ± 0.03	0.01 ± 0.02
714.5	PE (34:3)	2.20 ± 0.31	1.03 ± 1.64		6.53 ± 1.15	6.42 ± 2.00
716.5	PE (34:2)	17.99 ± 4.30	8.86 ± 5.59		43.63 ± 1.98	39.77 ± 2.23
718.5	PE (34:1)	4.06 ± 1.32	5.58 ± 2.39		0.43 ± 0.24	1.19 ± 0.93
736.5	PE (36:6)	0.14 ± 0.02	0.07 ± 0.07		1.03 ± 0.34	1.22 ± 0.12
738.5	PE (36:5)	2.35 ± 0.85	0.69 ± 1.19		9.29 ± 1.73	9.83 ± 1.48
740.5	PE (36:4)	20.60 ± 5.26	16.53 ± 6.56		30.17 ± 4.40	32.45 ± 2.23
742.5	PE (36:3)	12.20 ± 2.45	13.70 ± 3.05		1.59 ± 0.41	2.82 ± 1.01
744.5	PE (36:2)	21.18 ± 3.78	20.16 ± 1.83		0.77 ± 0.11	0.94 ± 0.22
746.6	PE (36:1)	3.41 ± 0.73	2.81 ± 2.43		0.00 ±0.00	0.00 ± 0.00
768.5	PE (38:4)	3.53 ± 4.76	3.65 ± 2.49		0.02 ± 0.02	0.02 ± 0.01
770.6	PE (38:3)	2.29 ± 1.35	2.09 ± 1.21		0.02 ± 0.03	0.05 ± 0.04

The PE species of which the proportions were less than 1% were removed (PE 32:3, PE 34:4, PE 36:6, PE 38:5, PE 38:6, PE 40:2, PE 40:3, PE 42:2, PE 42:3, PE 42:4).

**Table 4:** Classes and proportions of digalactosyl diacylglycerols in each sample (mol%).

		Hevea			TKS	
Mass	DGDG	Latex	WRP		Latex	WRP
932.6	DGDG (34:3)	2.60 ± 0.04	2.76 ± 1.15		11.39 ± 1.97	13.47 ± 1.92
934.6	DGDG (34:2)	5.48 ± 0.80	4.89 ± 1.07		14.87 ± 3.76	23.87 ± 6.50
936.6	DGDG (34:1)	4.68 ± 0.22	4.06 ± 0.61		1.56 ± 1.87	3.08 ± 2.28
954.6	DGDG (36:6)	0.72 ± 0.38	0.94 ± 0.66		20.30 ± 4.29	9.87 ± 9.91
956.6	DGDG (36:5)	5.22 ± 1.83	4.36 ± 1.90		28.79 ± 7.80	20.64 ± 3.08
958.6	DGDG (36:4)	22.08 ± 6.91	19.73 ± 8.16		19.16 ± 5.10	18.96 ± 2.85
960.6	DGDG (36:3)	20.90 ± 1.14	20.97 ± 1.90		2.45 ± 1.66	5.61 ± 4.41
962.6	DGDG (36:2)	21.79 ± 1.77	21.56 ± 2.49		1.14 ± 1.01	3.38 ± 4.91
964.7	DGDG (36:1)	10.98 ± 1.70	10.71 ± 1.79		0.15 ± 0.26	0.54 ± 0.93
986.6	DGDG (38:4)	2.15 ± 2.49	4.30 ± 3.41		0.10 ± 0.17	0.39 ± 0.48
988.7	DGDG (38:3)	2.70 ± 2.77	4.76 ± 3.53		0.00 ± 0.00	0.02 ± 0.03

The PC species of which the proportions were less than 1% were removed (PC 32:0, PC 34:4, PC 36:6, PC 38:2, PC 38:5, PC 38:6, PC 40:2, PC 40:3, PC 40:4, PC 40:5).

**Table 5:** Classes and proportions of monogalactosyl diacylglycerol in each sample (mol%).

		Hevea			TKS	
Mass	MGDG	Latex	WRP		Latex	WRP
770.5	MGDG(34:3)	1.39 ± 0.96	2.15 ± 0.22		1.14 ± 1.67	1.18 ± 1.02
772.6	MGDG(34:2)	2.41 ± 0.43	2.96 ± 0.36		0.32 ± 0.34	2.53 ± 3.66
774.6	MGDG(34:1)	1.56 ± 0.56	2.10 ± 0.24		0.00 ± 0.00	0.00 ± 0.00
792.5	MGDG(36:6)	1.87 ± 1.59	2.14 ± 2.5		22.65 ± 9.01	23.25 ± 22.52
794.5	MGDG(36:5)	9.80 ± 2.50	6.43 ± 1.91		38.88 ± 9.10	39.92 ± 14.45
796.6	MGDG(36:4)	34.71 ± 11.99	25.08 ± 11.54		36.96 ± 2.55	25.51 ± 5.20
798.6	MGDG(36:3)	20.08 ± 1.27	20.56 ± 2.45		0.00 ± 0.00	7.53 ± 9.66
800.6	MGDG(36:2)	13.32 ± 2.55	15.49 ± 2.78		0.00 ± 0.00	0.00 ± 0.00
802.6	MGDG(36:1)	6.25 ± 1.32	7.51 ± 1.47		0.00 ± 0.00	0.07 ± 0.13
824.6	MGDG(38:4)	3.11 ± 2.94	6.96 ± 3.92		0.00 ± 0.00	0.00 ± 0.00
826.6	MGDG(38:3)	5.16 ± 5.46	8.14 ± 4.88		0.00 ± 0.00	0.00 ± 0.00

The MGDG species of which the proportions were less than 1% were removed (MGDG 34:4, MGDG 34:5, MGDG 34:6, MGDG 38:5, MGDG 38:6).

**Table 6:** Proportions of compound lipid species in the latex and rubber particles (RP) of Hevea and TKS (mol% of total compound lipid).

	PC	PI	PE	PA	PG	DGDG	MGDG
**Hevea Latex**	70.7 ± 2.9	11.9 ± 1.6	2.1 ± 1.0	0.5 ± 0.4	0.1 ± 0.1	12.2 ± 1.2	1.3 ± 0.4
**Hevea RP**	69.7 ± 4.0	11.4 ± 5.3	2.1 ± 2.2	0.4 ± 0.3	0.1 ± 0.1	13.6 ± 1.6	2.1 ± 0.5
**TKS Latex**	75.4 ± 0.1	6.0 ± 0.3	14.3 ± 0.5	1.4 ± 0.2	1.1 ± 0.3	0.4 ± 0.1	0.4 ± 0.1
**TKS RP**	81.1 ± 4.2	3.1 ± 0.7	10.1 ± 2.7	3.2 ± 2.2	1.1 ± 0.3	0.2 ± 0.1	0.1 ± 0.1

The PS, LPC, LPE and LPG of which the proportions were less than 1% were removed.

## Supplementary Materials

The following are available online.
