# Supplementary material for: Lipid Composition of Latex and Rubber Particles in Hevea brasiliensis and Taraxacum kok-saghyz
Source: Molecules. 2020 Nov 3;25(21):5110. doi: 10.3390/molecules25215110 (PMC7662343; doi:10.3390/molecules25215110)
Supplement: Supplementary file 1 [file molecules-25-05110-s001.pdf]

Electronic Supplementary Information

Lipid composition of fresh latex and rubber particles in *Hevea brasiliensis* and *Taraxacum kok-saghyz*

Sung Woo Bae [1,2,3,†], Sunghee Jung [1,2,†], Sang Chul Choi [1], Mi Young Kim [1], and Stephen Beungtae Ryu [1,2,#]

[1] Plant Systems Engineering Research Center, Korea Research Institute of Bioscience & Biotechnology (KRIBB), Daejeon 34141, South Korea

[2] Department of Biosystems and Bioengineering, KRIBB School of Biotechnology, University of Science and Technology (UST), Daejeon 34141, South Korea

[3] Research & Development Center, JICO Ltd., Daejeon 341486, South Korea

# Correspondence to: sbryu@kribb.re.kr; Tel.: +82-42-860-4295 (S.B.R.)

† These authors contributed equally to this work

Figure S1: HPLC-ELSD chromatogram of phospholipid standards.

Table S1: Classes and proportions of phosphatidic acids in each sample (mol %).

Table S2: Classes and proportions of phosphatidylglycerols in each sample (mol %).

Table S3: Classes and proportions of phosphatidylserines in each sample (mol %).

Table S4: Classes and proportions of lysophosphatidylcholines in each sample(mol %).

(a)

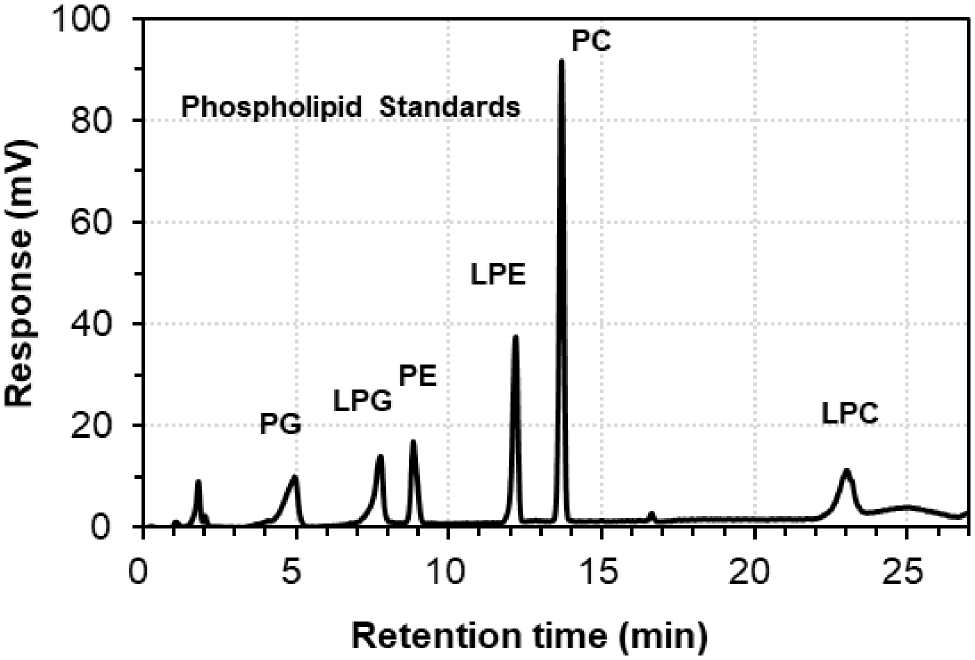

(b)

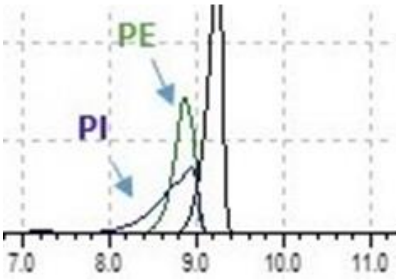

Figure S1: HPLC-ELSD chromatogram of phospholipid standards.

(**a**) Standards of PC(16:0/18:2), PE(16:0/18:2), PG(soy), LPC(18:1), LPE(18:1), and LPG(18:1); (**b**) Standards of PE(16:0/18:2) and PI(soy).

Table S1. Classes and proportions of phosphatidic acids in each sample (mol %).

		Hevea		TKS	
Mass	PA	Latex	WRP	Latex	WRP
666.5	PA(32:0)	3.63 ± 1.86	12.63 ± 10.22	0.00 ± 0.00	0.37 ± 0.43
688.5	PA(34:3)	9.53 ± 14.2	1.02 ± 1.77	8.52 ± 2.85	8.83 ± 4.23
690.5	PA(34:2)	13.54 ± 5.86	27.47 ± 13.49	63.10 ± 3.82	52.08 ± 4.11
692.5	PA(34:1)	5.63 ± 5.25	9.15 ± 4.78	5.13 ± 0.02	5.71 ± 2.04
712.5	PA(36:5)	2.49 ± 2.28	1.25 ± 2.17	5.23 ± 1.27	6.13 ± 0.69
714.5	PA(36:4)	33.51 ± 10.08	13.62 ± 8.87	15.77 ± 6.25	19.97 ± 3.16
716.5	PA(36:3)	13.39 ± 4.72	13.01 ± 12.02	1.80 ± 2.02	4.43 ± 1.22
718.5	PA(36:2)	18.03 ± 8.81	21.76 ± 10.85	0.46 ± 0.69	2.11 ± 2.21

* The PA species of which the proportions were less than 1% were removed (PA 34:4, PA 34:5, PA 34:6, PA 36:6)

Table S2. Classes and proportions of phosphatidylglycerols in each sample (mol %).

		Hevea		TKS	
Mass	PG	Latex	WRP	Latex	WRP
762.5	PG(34:3)	4.73 ± 3.09	3.95 ± 6.84	6.66 ± 2.84	7.17 ± 3.63
764.5	PG(34:2)	55.78 ± 14.50	56.02 ± 7.58	91.69 ± 2.21	88.50 ± 5.97
766.5	PG(34:1)	13.81 ± 12.31	17.32 ± 2.61	0.06 ± 0.10	2.52 ± 2.65
768.5	PG(34:0)	2.11 ± 1.85	0.25 ± 0.43	0.01 ± 0.01	0.00 ± 0.00
786.5	PG(36:5)	1.51 ± 1.31	10.26 ± 17.76	0.31 ± 0.35	0.34 ± 0.30
788.5	PG(36:4)	4.35 ± 3.98	9.13 ± 12.73	0.51 ± 0.24	1.21 ± 0.92
790.5	PG(36:3)	10.08 ± 17.45	2.28 ± 2.02	0.00 ± 0.00	0.24 ± 0.22
792.5	PG(36:2)	6.79 ± 6.69	0.00 ± 0.00	0.00 ± 0.00	0.04 ± 0.07

* The PG species of which the proportions were less than 1% were removed (PG 32:0, PG 32:1, PG 34:3, PG 36:1, PG 36:6)

Table S3. Classes and proportions of phosphatidylserines in each sample (mol %).

		Hevea		TKS	
Mass	PS	Latex	WRP	Latex	WRP
758.5	PS(34:3)	0.05 ± 0.09	0.00 ± 0.00	6.12 ± 2.65	5.18 ± 6.28
760.5	PS(34:2)	6.03 ± 0.73	3.08 ± 3.33	36.53 ± 8.63	28.17 ± 2.44
762.5	PS(34:1)	4.49 ± 1.86	2.64 ± 2.32	0.50 ± 0.23	1.32 ± 1.18
782.5	PS(36:5)	0.15 ± 0.27	0.00 ± 0.00	1.80 ± 0.76	1.49 ± 2.08
784.5	PS(36:4)	1.16 ± 0.79	0.48 ± 0.84	9.59 ± 3.44	13.46 ± 5.09
786.5	PS(36:3)	7.40 ± 3.14	8.69 ± 0.75	0.00 ± 0.00	2.67 ± 0.83
788.5	PS(36:2)	18.41 ± 3.05	26.69 ± 5.40	0.57 ± 0.98	2.92 ± 2.23
790.6	PS(36:1)	2.06 ± 2.24	1.42 ± 2.46	0.00 ± 0.00	0.00 ± 0.00
812.5	PS(38:4)	1.92 ± 1.04	0.94 ± 1.62	0.19 ± 0.18	0.24 ± 0.42
814.6	PS(38:3)	7.84 ± 1.30	6.25 ± 2.93	0.24 ± 0.42	3.04 ± 1.38
816.6	PS(38:2)	23.09 ± 2.19	31.66 ± 9.41	10.10 ± 4.97	10.02 ± 2.77
818.6	PS(38:1)	9.10 ± 4.32	9.50 ± 2.80	0.00 ± 0.00	3.07 ± 3.28
842.6	PS(40:3)	1.47 ± 1.31	0.51 ± 0.89	5.57 ± 2.35	4.43 ± 3.13
844.6	PS(40:2)	10.76 ± 1.82	4.76 ± 2.33	25.66 ± 7.35	18.84 ± 5.14
846.6	PS(40:1)	5.42 ± 1.14	3.16 ± 3.21	0.00 ± 0.00	2.12 ± 1.99
872.6	PS(42:2)	0.27 ± 0.47	0.00 ± 0.00	2.93 ± 3.65	2.67 ± 2.82

* The PS species of which the proportions were less than 1% were removed (PS 34:4, PS 36:6, PS 38:5, PS 38:6, PS 40:4, PS 42:1, PS 42:3, PS 42:4, PS 44:2, PS 44:3)

Table S4. Classes and proportions of lysophosphatidylcholines in each sample (mol %).

Mass	LPC	Hevea		TKS	
		Latex	WRP**	Latex	WRP
496.3	LPC(16:0)	5.27 ± 2.93	15.83 ± 13.12	24.82 ± 0.76	21.46 ± 2.64
518.3	LPC(18:3)	4.27 ± 0.42	4.34 ± 2.96	11.13 ± 3.61	12.3 ± 0.8
520.3	LPC(18:2)	57.89 ± 4.99	47.07 ± 9.66	58.38 ± 3.64	56.76 ± 3.38
522.3	LPC(18:1)	22.15 ± 3.15	20.61 ± 10.04	1.42 ± 2.46	7.71 ± 4.83
524.4	LPC(18:0)	10.02 ± 2.62	11.28 ± 3.31	4.25 ± 6.91	1.58 ± 1.5

* The LPC species of which the proportion was less than 1% was removed (LPC 16:1)
